# A Novel High-Speed and High-Accuracy Mathematical Modeling Method of Complex MEMS Resonator Structures Based on the Multilayer Perceptron Neural Network

**DOI:** 10.3390/mi12111313

**Published:** 2021-10-26

**Authors:** Qingsong Li, Kuo Lu, Kai Wu, Hao Zhang, Xiaopeng Sun, Xuezhong Wu, Dingbang Xiao

**Affiliations:** 1 College of Intelligence Science and Technology, National University of Defense Technology, Changsha 410073, China; liqingsong12@nudt.edu.cn (Q.L.); lukuo13@nudt.edu.cn (K.L.); wukai@nudt.edu.cn (K.W.); zhanghao19@nudt.edu.cn (H.Z.); sxp@nudt.edu.cn (X.S.); xzwu@nudt.edu.cn (X.W.); 2 Hunan MEMS Research Center, Changsha 410073, China; 3 Laboratory of Science and Technology on Integrated Logistics Support, National University of Defense Technology, Changsha 410073, China

**Keywords:** mathematical modeling, finite element analysis, artificial neural network, multilayer perceptron neural network, structural optimization design

## Abstract

MEMS resonators have become core devices in a large number of fields; however, due to their complex structures, the finite element analysis (FEA) method is still the main method for their theoretical analysis. The traditional finite element analysis method faces the disadvantages of large calculation amount and long simulation time, which limits the development of high-performance MEMS resonators. This paper demonstrates a high-speed and high-accuracy simulation tool based on the artificial neural network, where a multilayer perceptron (MLP) neural network model is constructed. The typical structural parameters of MEMS resonator are used as the input layer, and its performance indicators produced by the finite element analysis method are the output layer. After iteratively trained with 4000 samples, the cumulative error of the neural network decreases to 0.0017 and a prediction network model is obtained. Compared with the finite element analysis results, the structural accuracy error predicted by the neural network model can be controlled within 6%, but its runtime is shortened by 15,000 times. This high-speed and high-accuracy mathematical modeling method can effectively improve the analyzing efficiency and provide a promising tool for the design and optimization of different complex MEMS resonators, which exhibit remarkable accuracy and speed.

## 1. Introduction

With the development of the modern technology, micro-electro-mechanical system (MEMS) devices have become an important branch of the core sensor field [1,2,3,4,5,6,7,8,9]. Due to its advantages of small size, low cost and mass production, MEMS devices have attracted the attention of many research teams and commercial companies, dramatically increasing their demands [10,11]. Among these devices, the structure of MEMS resonators is the core, which determines their performance. Therefore, analyzing and evaluating the performance of MEMS resonator structures is a key step to promote the application and development of MEMS devices, which is also the direction that many research teams are committed to study and has been reported in many literatures [12]. However, the complex topological structure and numerous parameters make this research particularly difficult.

According to previous research, the performance of the MEMS resonators is usually obtained by finite element analysis method with related simulation software. The finite element analysis is a numerical solution method that uses mathematical approximations to simulate real physical systems [13,14]. However, as for a complex MEMS resonator structure, under the combined influence of topological structure and mesh density, the finite element analysis method will take a long time to do the simulation, which severely limits the progress rate of structural design. As a result, the development of a novel high-speed and high-accuracy mathematical modeling method for complex MEMS resonator structures is urgent.

The artificial neural network (ANN) is an algorithmic mathematical model that imitates the behavioral characteristics of animal neural networks and performs distributed and parallel information processing [15,16,17]. It has preliminary self-adaptation and self-organization capabilities. In the learning or training process, by changing synaptic weight values, this model can be adapted to the requirements of the environment. As a result, the artificial neural network and the finite element analysis method can be combined flexibly. The artificial neural network model is trained on the basis of an appropriate amount of finite element analysis result dataset, so that it can accurately predict the finite element analysis process and provide an efficient and convenient simulation tool. In this case, it can effectively replace the long-term finite element simulation process and improve the efficiency of the analysis.

In this paper, a high-speed and high-accuracy method based on the multilayer perceptron neural network is put forward to mathematically simulate complex MEMS resonator structures. The disk MEMS resonator is taken as the research object. It is a complex topological structure determined by multiple structural parameters, with typical representativeness and research value. In the following sections, the basic structural parameters and performance indicators of the disk MEMS resonator are analyzed. At the same time, the finite element analysis method is introduced and used to simulate the disk MEMS resonators’ performance, to provide enough training and testing data. Then, the theoretically analysis of the multilayer perceptron neural network and its model structure are studied. Furthermore, the structural performance indicators learned by the neural network according to the prepared dataset are compared with the finite element analysis results. Finally, the basic principles for the application of the multilayer perceptron neural network in MEMS resonators are concluded.

## 2. Disk MEMS Resonator

### 2.1. Structure Description

**Figure 1:**
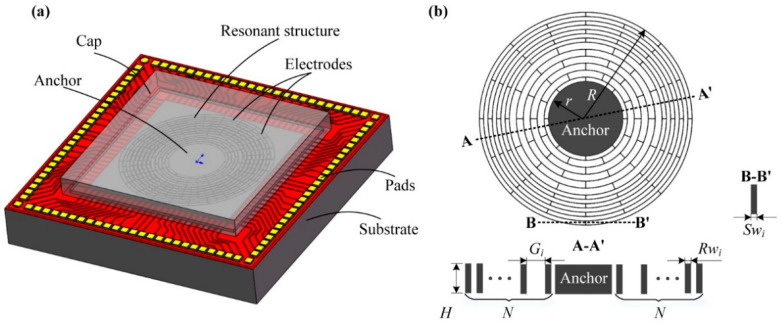
The schematic diagram of the disk MEMS resonator. (**a**) The wafer-level disk MEMS resonator; (**b**) the resonant structure of the disk MEMS resonator.

The disk MEMS resonator is a typical distributed mass structure, which is composed of multiple connected beams and rings [18,19]. Its key structural parameters include height, spoke width, ring width, ring gap, anchor radius and ring number, which contribute to its final performance indicators. Numerous structural parameters make the structure of the disk MEMS resonator relatively complex and hard to be modeled. The structure of a typical wafer-level disk MEMS resonator is shown in Figure 1a, which is mainly composed of a substrate, an oxidizing isolation layer, a resonant structure, and multiple electrodes [20]. The silicon-based substrate is used to carry the resonant structure and electrodes. Its oxidation isolation layer is used to be insulated. The resonant structure is the core component of the resonator, as shown in Figure 1b, which is mainly connected by a plurality of concentric rings and spokes. The gaps between those rings are used to place electrodes to drive, detect and tune the disk MEMS resonator.

Obviously, the structure of the disk MEMS resonator is relatively complicated, with numerous structural parameters. In order to facilitate the subsequent analysis, the main structural parameters are defined as follows: the thickness of the structure layer *H*, the number of resonant rings *N*, the ring thickness *Rw*, the gap between rings (spoke length) *G*, the radius of the anchor *r*, and the width of the spoke *Sw*. It is worth noting that because the structure of disk MEMS resonator is composed of multiple nested rings, a typical structure contains multiple *Rw*, *G*, and *Sw*. These values at the position of the *i*th ring can be expressed as *Rw_i_*, *G_i_*, and *Sw_i_*, where *i* = 1, 2, …, *N*; these values can be different from each other. Through these parameters, a typical disk MEMS resonator can be completely designed.

In addition, it is reasonable that the structural parameters of disk MEMS resonators are not unlimited. According to prior knowledge and previous experience, these structural parameters can be constrained in reasonable value ranges. Using values in the interval for structural design can be purposeful and directional, and can effectively reduce the number of trial and error. The value ranges of different structural parameters of the disk MEMS resonator used in the design process of this paper are shown in Table 1.

The complex structure and numerous parameters pose a huge challenge to the disk MEMS resonator’s design and analysis process. To achieve a reasonable design and improve the resonator’s performance, an accurate performance evaluation is required.

### 2.2. Core Performance Indicators

The characteristics of the resonant structure determine the ultimate performance of the MEMS resonator. As a typical representative, the performance of a disk MEMS resonator can be mainly characterized by its mechanical structure performance indicators, thermodynamic performance indicators, and electrical performance indicators [12].

#### 2.2.1. Fundamental Frequency

The disk MEMS resonator is a typical representative of MEMS sensors that works in a specific operating mode [21]. The fundamental frequency of the operating mode is one of the core performance indicators. It is mainly determined by the resonator’s material properties and structural characteristics. When the disk MEMS resonator is excited by the resonance frequency of its operating mode, its dynamic response can be amplified and its sensitivity can be improved. For a common traditional mass-stiffness system, its resonant frequency can be calculated as:

(1) $$f_{i}=\sqrt{\frac{k_{i}}{m_{i}}}$$

#### 2.2.2. Quality Factor

In addition, the quality factor (Q) is the key indicator to evaluate the dynamic characteristics of the resonator, and a resonator with a higher quality factor will have better performance characteristics. It is an evaluation of the energy dissipation and damping characteristics in the micromechanical resonator system, which is mainly affected by the damping loss. The energy of the resonator will be dissipated in the form of heat during a period of movement. There are different energy loss mechanisms in MEMS resonators, including thermoelastic damping, air damping, anchor loss, surface loss, etc. For the disk MEMS resonator, in order to improve its vibration stability and reduce the power consumption of the system, it is generally expected that it has a higher quality factor characteristic. It can be obtained from Equation (2) that the total quality factor is limited to the smallest one of its many quality factors:

(2) $$\frac{1}{Q}=\frac{1}{Q_{Thermoelastic}}+\frac{1}{Q_{air}}+\frac{1}{Q_{anchor}}+\frac{1}{Q_{surface}}+\frac{1}{Q_{other}}$$

For the disk MEMS resonator, due to its symmetrical planar structure, its support loss can be neglected; at the same time, since the MEMS resonator usually adopts a mature high-vacuum packaging process, its air loss can also be controlled in a very small range. Therefore, the thermoelastic damping is the key factor, which could be explained by the Zener’s formalism [22]. It is mainly determined by the resonant structure parameters and material properties [23,24]. Therefore, in order to improve the theoretical quality factor of the disk MEMS resonator, optimizing its geometry and size is a common method to reduce the thermoelastic damping.

#### 2.2.3. Mechanical Sensitivity

The output of the disk MEMS resonator contains effective signals and error signals, so that one of the designing goals is to improve the resonator’s signal-to-noise ratio, which could be described by the mechanical sensitivity. The mechanical sensitivity is an important parameter that characterizes the resolution of the detection axis displacement of the resonator in the force feedback control mode. It determines the accuracy of the resonator system. The expression of the mechanical sensitivity of the classic disk MEMS resonator can be expressed in Equation (3) [25]:

(3) $$S_{mech}=\frac{2k\left|\bar{x}\right|}{\sqrt{{\left(\frac{\omega_{0}^{2}}{\omega_{d}}-\omega_{d}\right)}^{2}+\frac{4}{\tau_{0}^{2}}}}$$

where *k* is the Coriolis coupling coefficient, $\bar{x}$ is the amplitude of the resonator when it is working, *ω*_0_ is the natural frequency of the working mode, *ω_d_* is the frequency of the driving signal, and *τ*_0_ is the decay time constant of the working mode, which is related to the quality factor of the resonator.

#### 2.2.4. Mechanical Thermal Noise

The resolution of the disk MEMS resonator is mainly determined by the mechanical thermal noise and the circuit noise. The mechanical thermal noise determines the limit resolution of the disk MEMS resonator and is closely related to its structural parameters and material properties. The mechanical thermal noise is random motion caused by the Brownian thermal motion of the MEMS resonator structure itself, the expression of which is shown in Equation (4) [26]:

(4) $$\Omega_{Brown}=\frac{1}{k\left|\bar{x}\right|}\sqrt{\frac{2k_{B}T}{k_{eff}\tau_{0}}}$$

where *k_B_* is the Boltzmann’s constant, *T* is the absolute temperature, and *k_eff_* is the equivalent stiffness coefficient of the working mode of the resonator.

In summary, the core performance indicators of the disk MEMS resonant gyroscope studied in this paper are shown in Table 2.

### 2.3. Finite Element Analysis Method

In the designing process, it is necessary to obtain theoretical performance indicators of the disk MEMS resonator. These performance indicators can be used to obtain feedback and optimize the structural design parameters. Therefore, the finite element analysis method is applied to provide related simulation research.

The basic idea of the finite element analysis method is to discretize the structure and use a finite number of easy-to-analyze elements to represent complex objects. The elements are connected to each other through a finite number of nodes, and then they are comprehensively solved according to the deformation coordination conditions. However, the meshing density has a great influence on the accuracy of simulating results. The higher accuracy of finite element analysis, the more computing time and computing resources are required.

**Figure 2:**
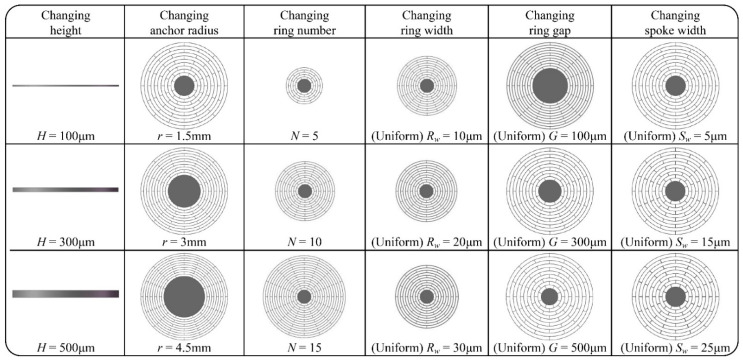
Typical structure examples of the disk MEMS resonator with only one single structural parameter changed.

In this paper, the finite element analysis simulation is used to provide enough simulation results for different disk MEMS resonators, which will be used as the training dataset and testing dataset. To make samples more representative and disorderly, the structural parameters of the disk resonator studied in the paper are randomly selected from the corresponding value range, and then simulated by simulation software to extract its performance indicators. In order to visually show the changes in disk MEMS resonators’ structures, some typical disk resonator structures with only one single structural parameter changed are selected and displayed in Figure 2. In the actual simulation process, the structural parameters of the disk resonator are changed together by multiple parameters, and are not limited to changing only one single parameter. At the same time, their changing order and trend are also random.

## 3. Multilayer Perceptron Neural Network Model

A neural network is a widely parallel interconnected network composed of adaptable simple units [27]. According to the input of the system, by constructing a neural network structure and adjusting the distribution weight of neurons and the number of propagation layers, the corresponding system response output can be obtained. Based on the characteristics of the neural network, it can be applied to other fields of engineering and scientific research.

**Figure 3:**
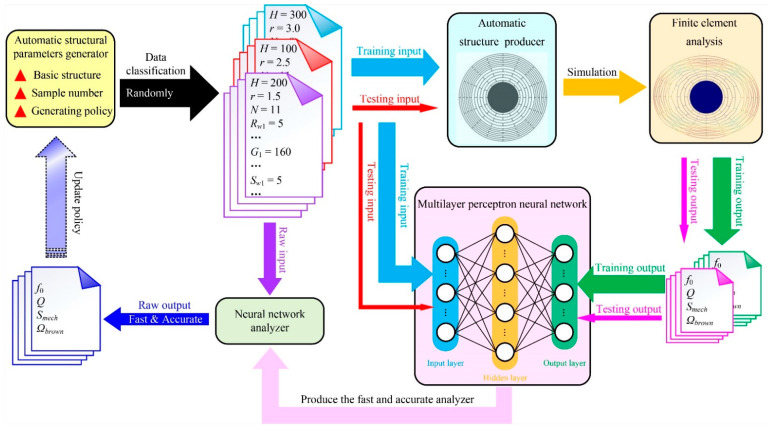
The top-level architecture for the high-speed and high-accuracy disk MEMS resonator simulation analyzer enabled by the multilayer perceptron neural network.

Through extensive training datasets, the neural network structure is optimized, and the ideal output value can be reasonably predicted based on the input value. Therefore, proper neural network structure parameters are the key to the high speed and high accuracy of prediction. Due to the complex structure, it is extremely difficult to manually modify and optimize its structure parameters, and machine learning methods can be used to optimize it [28]. The machine learning method can effectively improve the optimization efficiency, greatly reduce the optimization time, and realize the rapid convergence of the neural network structure. It is a typical regression task using the neural network to learn the simulation process of the finite element analysis method. Therefore, based on the neural network and machine learning method, this paper proposes a high-speed and high-accuracy simulation analyzer, and its top-level architecture is shown in Figure 3.

### 3.1. Dataset Definition

Machine learning is an algorithm that uses a large amount of historical data to dig out the hidden laws, which could be used for prediction or classification [27]. The generalization error of the learning algorithm could be evaluated through an experimental test. The overall dataset can be divided into two mutually exclusive sets. One set is used as the training set ***S*** to guide the update iteration of the learning algorithm; the other set is used as the test set ***T*** to test the learning algorithm’s ability to analyze new samples. In this case, the basic idea is to first train the model within the training set ***S***, and then verify it on the test set ***T***, and evaluate the test error as an estimate of the generalization error.

In order to ensure the validity of the dataset, it is necessary to maintain the consistency of the data distribution when dividing the training set and the test set. This paper adopts a dual random classification method: firstly, in the process of generating the parameters of the dataset, the method of randomly picking values in the parameter interval is used to generate the dataset; then, when dividing the training set and the test set, the random sampling method is used to divide. These methods can effectively reduce the subjective error introduced when the dataset is classified, and ensure the uniformity and consistency of the dataset distribution. In this case, the additional bias introduced by the input division process can be avoided.

In this paper, we randomly generated 4000 sets of data, and randomly selected 3200 sets of data as the training set for the machine learning on the neural network structure; the remaining 800 sets of data were used as the test set to detect neural network models’ accuracy and reliability.

### 3.2. Multilayer Perceptron Neural Network

The multilayer perceptron neural network is also called the artificial neural network, which introduces one or more hidden layers on the basis of a single-layer neural network [27,29]. In addition to the input and output layers, there are multiple hidden layers in the middle. The simplest MLP model only contains one hidden layer, which is a three-layer structure. It is a composite architecture composed of multiple neurons in an orderly distribution. Its core mainly includes two parts: a feedforward neural network and an error back propagation algorithm. The following subsections will systematically describe the multilayer perceptron model constructed in this paper.

#### 3.2.1. M-P Neuron Model

**Figure 4:**
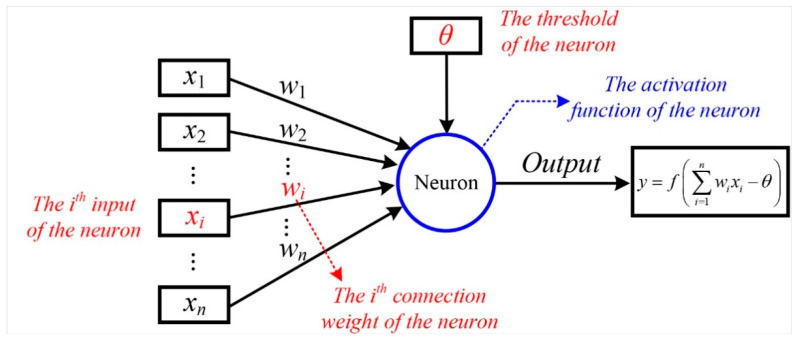
The M-P neuron model.

A neural network is a composite structure composed of multiple neurons, and its basic unit is a neuron [27]. The used neuron model in this paper is the M-P neuron model proposed by McCulloch and Pitts [30], which could be described in Figure 4. In this model, the neuron receives input signals from *n* other neurons, and these input signals are transmitted through weighted connections. The total input value received by the neuron will be compared with the neuron’s threshold *θ*, and then processed through the activation function *f(·)* to produce the neuron’s output.

In our model, the output of a single neuron can be expressed as:

(5) $$y=f\left(\sum_{i=1}^{n}w_{i}x_{i}-\theta \right)$$

where *x_i_* and *w_i_* (*i* = 1, 2, 3, …, n) are the inputs of the neuron and the weight coefficients, respectively. *θ* is the threshold of the neuron and *f(·)* is its activation function, which is used to process the input signal of the neuron to generate its output signals.

However, since the structural analysis of the MEMS resonator is a complex nonlinear problem, this simple linear model is obviously limited. Therefore, in order to make the model better reflect the actual condition, the Sigmoid function is taken as the activation function in this paper, which can make the neural network arbitrarily approximate any nonlinear function [28]. The mathematical expression of Sigmoid activation function is as follows:

(6) $$sigmoid\left(x\right)=\frac{1}{1+e^{-x}}$$

#### 3.2.2. Multi-Layer Feedforward Neural Networks

Generally, it is believed that the typical structural parameters of disk MEMS resonators are independent of each other and do not affect each other; at the same time, all structural parameters have an impact on the final performance indicators of the resonator. As a result, the multilayer perceptron architecture is applied in this paper. The multilayer perceptron model is a typical multilayer feedforward neural network. In this neural network structure, neurons in each layer are fully interconnected with neurons in the next layer, indicating that the neurons in the bottom layer all contribute to the state of the neurons in the next layer; there is no connection between neurons in the same layer, indicating that neurons in the same layer are independent of each other and do not affect each other; at the same time, there is no cross-layer connection.

**Figure 5:**
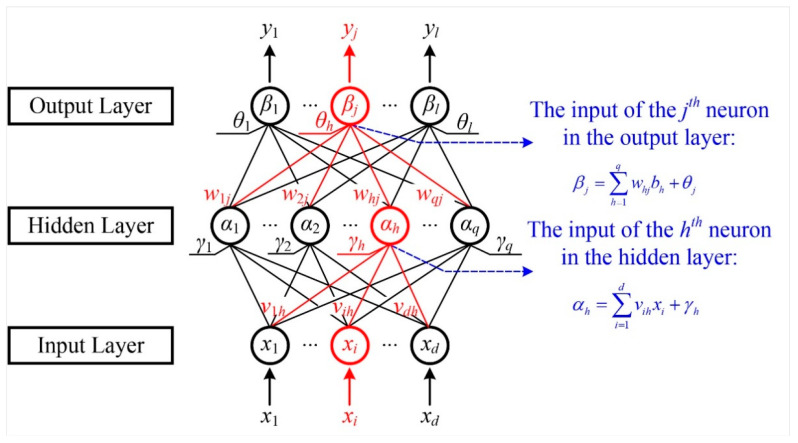
The forward propagation schematic diagram of the three-layer multilayer perceptron model.

The first step of the multilayer perceptron architecture is to transfer the signal from the input layer to the output layer, which is the process of forward propagation. Forward propagation refers to the process of gradually transferring information from the first layer to the high-level layer. The forward propagation schematic diagram of the three-layer multilayer perceptron model is shown in Figure 5.

Figure 5 exhibits the three-layer feedforward propagation network structure used in this paper, which contains *d* input neurons, *q* hidden neurons, and *l* output neurons. The initial input information of the model is [*x_1_*, *x_2_*, …, *x_d_*], and the final output information is [*y_1_*, *y_2_*, …, *y_l_*]. In this model, the threshold of the *h*th neuron in the hidden layer is represented by *γ_h_*, and the threshold of the *j*th neuron in the output layer is represented by *θ_j_*. In addition, the connection weight between the *i*th neuron in the input layer and the *h*th neuron in the hidden layer is *v_ih_*, and the connection weight between the *h*th neuron in the hidden layer and the *j*th neuron in the output layer is *w_hj_*. The activation function of the hidden layer is *f(·)* and the activation function of the output layer is *g(·)*. Therefore, the input signal received by the *h*th neuron in the hidden layer is shown in Equation (7):

(7) $$\alpha_{h}=\sum_{i=1}^{d}v_{ih}x_{i}$$

The input signal received by the *j*th neuron in the output layer is as follows:

(8) $$\beta_{j}=\sum_{h=1}^{q}w_{hj}b_{h}$$

Among them, *b_h_* is the output of the *h*th neuron in the hidden layer, which can be expressed as:

(9) $$b_{h}=f\left(\alpha_{h}-\gamma_{h}\right)=f\left(\sum_{i=1}^{d}v_{ih}x_{i}-\gamma_{h}\right)$$

As a result, the final output signal of the *j*th neuron in the output layer of the perceptron can be expressed as:

(10) $$y_{j}=g\left(\beta_{j}-\theta_{j}\right)=g\left(\sum_{h=1}^{q}w_{hj}b_{h}-\theta_{j}\right)=g\left[\sum_{h=1}^{q}w_{hj}\cdot f\left(\sum_{i=1}^{d}v_{ih}x_{i}-\gamma_{h}\right)-\theta_{j}\right]$$

Finally, the matrix expression of the forward propagation model of the entire three-layer perceptron can be obtained:

(11) $$\begin{array}{ll} y & =\left[\begin{array}{c} y_{1}\\ \vdots \\ y_{j}\\ \vdots \\ y_{l} \end{array}\right]=g\left(\left[\begin{array}{ccccc} w_{11} & \cdots & w_{1h} & \cdots & w_{1q}\\ \vdots & \ddots & \vdots & \ddots & \vdots \\ w_{j1} & \cdots & w_{jh} & \cdots & w_{jq}\\ \vdots & \ddots & \vdots & \ddots & \vdots \\ w_{l1} & \cdots & w_{lh} & \cdots & w_{lq} \end{array}\right]\left[\begin{array}{c} b_{1}\\ \vdots \\ b_{h}\\ \vdots \\ b_{q} \end{array}\right]-\left[\begin{array}{c} \theta_{1}\\ \vdots \\ \theta_{j}\\ \vdots \\ \theta_{l} \end{array}\right]\right)=g\left(w\left[\begin{array}{c} b_{1}\\ \vdots \\ b_{j}\\ \vdots \\ b_{q} \end{array}\right]-\theta \right)\\ & =g\left(w\cdot f\left(\left[\begin{array}{ccccc} v_{11} & \cdots & v_{1i} & \cdots & v_{1d}\\ \vdots & \ddots & \vdots & \ddots & \vdots \\ v_{h1} & \cdots & v_{hi} & \cdots & v_{hd}\\ \vdots & \ddots & \vdots & \ddots & \vdots \\ v_{q1} & \cdots & v_{qi} & \cdots & v_{qd} \end{array}\right]\left[\begin{array}{c} x_{1}\\ \vdots \\ x_{i}\\ \vdots \\ x_{d} \end{array}\right]-\left[\begin{array}{c} \gamma_{1}\\ \vdots \\ \gamma_{h}\\ \vdots \\ \gamma_{q} \end{array}\right]\right)-\theta \right)=g\left(w\cdot f\left(vx-\gamma \right)-\theta \right) \end{array}$$

#### 3.2.3. Error Back Propagation Algorithm

Another core task is updating the multilayer perceptron model’s structural parameters, where the error back propagation algorithm is applied in this paper. The back propagation of the loss function from the top layer to the bottom layer was used to realize the updating optimization of these parameters [27,31]. In this model, the mean squared error characterizes the degree of difference between the predicted value of the neural network and the theoretical value, which is used to express the learning accuracy of the multilayer perceptron neural network. According to Equation (5), for the training example (***x****_k_*, ***y****_k_*), the output of the neural network can be expressed as:

(12) $${\hat{y}}_{k}=\left({\hat{y}}_{1}^{k},{\hat{y}}_{2}^{k},\cdots ,{\hat{y}}_{l}^{k}\right)$$

where:

(13) $${\hat{y}}_{j}^{k}=f\left(\beta_{j}-\theta_{j}\right),j=1,2,\cdots l$$

Therefore, the mean squared error of this neural network on the training set (***x****_k_*, ***y****_k_*) is as follows:

(14) $$E_{k}=\frac{1}{2}\sum_{j=1}^{l}{\left({\hat{y}}_{j}^{k}-y_{j}^{k}\right)}^{2}$$

In the three layers perceptron neural network designed in this paper, as shown in Figure 5, a total of *(d + l + 1) × q + l* network parameters need to be optimized: *d × q* weight values from the input layer to the hidden layer, *q × l* weight values from the hidden layer to the output layer, *q* thresholds of hidden layer neurons, and *l* thresholds of output layer neurons.

The error back propagation algorithm used in this paper is based on a gradient descent strategy, which optimizes the parameters in the direction of the negative gradient of the target. The learning rate *η* ∈ (*0, 1*) controls the update step size in each iteration of the algorithm. For the connection weight *w_hj_* from the hidden layer to the output layer, when the learning rate is *η*, its updated value can be expressed as:

(15) $$\Delta w_{hj}=-\eta \frac{\partial E_{k}}{\partial w_{hj}}$$

According to the forward propagation network model, it is obvious that *w_hj_* first affects the input value *β_j_* of the *j*th output layer neuron, then affects its output value ${\hat{y}}_{j}^{k}$, and finally affects *E_k_*, so:

(16) $$\frac{\partial E_{k}}{\partial w_{hj}}=\frac{\partial E_{k}}{\partial {\hat{y}}_{j}^{k}}\cdot \frac{\partial {\hat{y}}_{j}^{k}}{\partial \beta_{j}}\cdot \frac{\partial \beta_{j}}{\partial w_{hj}}$$

Combined with Equation (8), it can be seen that:

(17) $$\frac{\partial \beta_{j}}{\partial w_{hj}}=b_{h}$$

In this paper, the activation functions of both the hidden layer and the output layer adopt the Sigmoid function, which has a good property to satisfy the following derivative:

(18) $$f^{\prime }\left(x\right)=f\left(x\right)\left(1-f\left(x\right)\right)$$

On this basis, combining Equations (13) and (14), we obtain:

(19) $$g_{j}=-\frac{\partial E_{k}}{\partial {\hat{y}}_{j}^{k}}\cdot \frac{\partial {\hat{y}}_{j}^{k}}{\partial \beta_{j}}=-\left({\hat{y}}_{j}^{k}-y_{j}^{k}\right)f^{\prime }\left(\beta_{j}-\theta_{j}\right)={\hat{y}}_{j}^{k}\left(1-{\hat{y}}_{j}^{k}\right)\left(y_{j}^{k}-{\hat{y}}_{j}^{k}\right)$$

Substituting Equations (17) and (19) into Equation (16), and then substituting the result into Equation (15), the updated equation for *w_hj_* in the error back propagation algorithm can be expressed as:

(20) $$\Delta w_{hj}=\eta g_{j}b_{h}$$

In the same way, other updated equations can be obtained:

(21) $$\Delta \theta_{j}=-\eta g_{j}$$

(22) $$\Delta v_{ih}=\eta e_{h}x_{i}$$

(23) $$\Delta \gamma_{h}=-\eta e_{h}$$

where:

(24) $$e_{h}=-\frac{\partial E_{k}}{\partial b_{h}}\cdot \frac{\partial b_{h}}{\partial \alpha_{h}}=-\sum_{j=1}^{l}\frac{\partial E_{k}}{\partial \beta_{j}}\cdot \frac{\partial \beta_{j}}{\partial b_{h}}f^{\prime }\left(\alpha_{h}-\gamma_{h}\right)=\sum_{j=1}^{l}w_{hj}g_{j}f^{\prime }\left(\alpha_{h}-\gamma_{h}\right)=b_{h}\left(1-b_{h}\right)\sum_{j=1}^{l}w_{hj}g_{j}$$

As for the multilayer perceptron neural network, the goal of the error back propagation algorithm is to minimize the cumulative error on the training set:

(25) $$E=\frac{1}{m}\sum_{k=1}^{m}E_{k}$$

Therefore, the cumulative error back propagation algorithm gets the errors of the entire training set before updating and optimizing the parameters. The working flow chart of the error back propagation algorithm used in this paper is displayed in Table 3. For each training example, the error back propagation algorithm performs the following operations: (1) first provide the input example to the input layer neuron, and then forward the signal layer by layer until the output layer result is produced; (2) calculate the output layer’s error (corresponding to Process 4 to Process 5 in the Table 3), and then propagate the error back to the hidden layer neuron (corresponding to Process 6 in the Table 3); (3) finally, adjust the connection weights and thresholds based on the error of the hidden layer neuron (corresponding to Process 7 in the Table 3). This iterative process is repeated until the stopping condition is satisfied. The stop condition used in this paper is that the cumulative error is less than 10^−3^ or the number of iterations exceeds 10^5^.

## 4. Discussion

### 4.1. Neural Network Structure Parameters

In this paper, a traditional three-layer perceptron neural network as the deep learning tool to build a high-speed and high-accuracy mathematical modeling analysis model is explored. In this neural network structure, the number of the input layer’s neurons *d* is equal to the number of the typical disk MEMS resonator’s basic structural parameters, while the number of the output layer’s neurons *l* is equal to the number of the resonator’s core performance indicators. Considering the complexity and accuracy of the model, the number of neurons in the hidden layer of the neural network built in this paper is set as (*d* + *l*) × 30 [27,32]. The learning rate is 0.01 and the activation functions are both Sigmoid functions. The stop condition of the training process is that its cumulative error is less than 10^−3^ or the number of iterations exceeds 10^5^.

### 4.2. Neural Network Learning Performance

**Figure 6:**
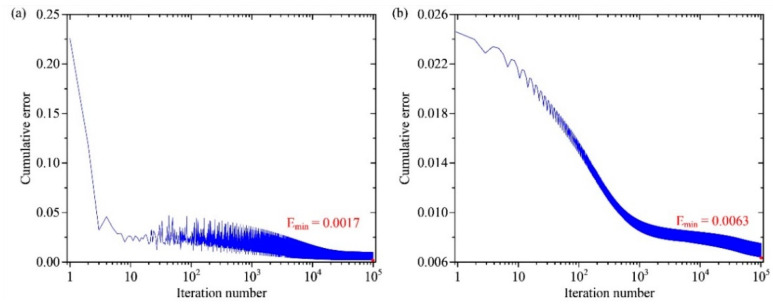
The cumulative error curves of the multilayer perceptron neural network. (**a**) The cumulative error curve of the training dataset; (**b**) the cumulative error curve of the test dataset.

The multilayer perceptron neural network needs to be trained before performing the prediction process. The automatic structural parameters generator produces 4000 different typical disk MEMS resonator samples, which are divided as the training dataset and the testing dataset. With these sample data, the multilayer perceptron neural network steps into the training process. To obtain an effective mathematical modeling tool with high speed and high accuracy, the entire training process takes about 516 min by using an NVIDIA GeForce GTX 1660Ti graphics cards. With more advanced equipment, both the training time and the runtime can be potentially further reduced. The cumulative error curves of the multilayer perceptron neural network are displayed in Figure 6. It is obvious that with the increasing of the iterations, the cumulative errors of the training data and the testing data both decrease gradually, which are 0.0017 and 0.0063 in the end, respectively. It proves that the cumulative error of the multilayer perceptron neural network reaches a satisfying level.

Generally, the evaluation indicators of the neural network learning performance are the prediction speed and the prediction accuracy. In the evaluating process, the performance of our high-speed and high-accuracy mathematical modeling tool generated by the multilayer perceptron neural network is compared with the corresponding performance produced by the traditional finite element analysis, which is set as the reference.

**Figure 7:**
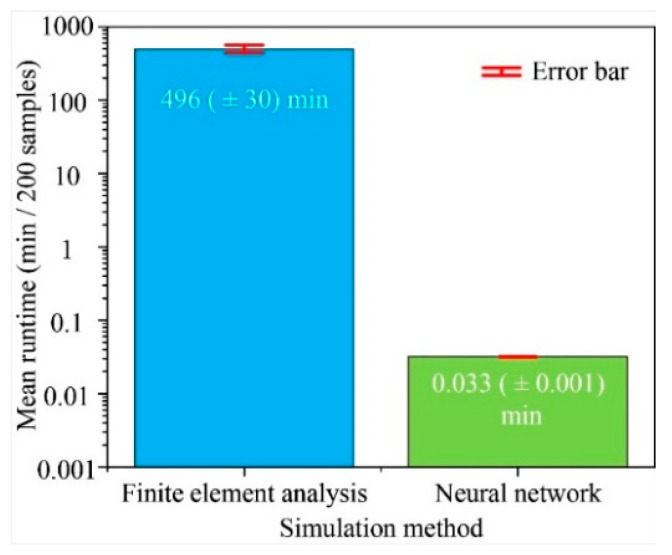
Runtime comparison between the traditional finite element analysis method and the neural network analyzer based on the multilayer perceptron neural network.

In order to fairly compare the runtime of different methods, these two methods dealt with the same structural models under the same computer configuration. Firstly, the automatic structural parameters generator produces 800 new typical disk MEMS resonator’s structural parameters. Then, these two methods both simulates or predicts these samples’ performance indicators. The runtime for 200 samples comparison between the traditional finite element analysis method and the neural network analyzer based on the multilayer perceptron neural network is shown in Figure 7. The average runtime for the traditional finite element analysis method is 496 (±30) minutes per 200 samples, while the time consumption of the neural network for the same work is only 0.033 (±0.001) minutes. It is obvious that the comparation speed of the neural network is much higher than the traditional finite element analysis speed. The calculation speed of the neural network is nearly about 15,000 times that of the finite element analysis speed, exhibiting excellent calculation characteristics and effectively shortening the simulation time.

**Figure 8:**
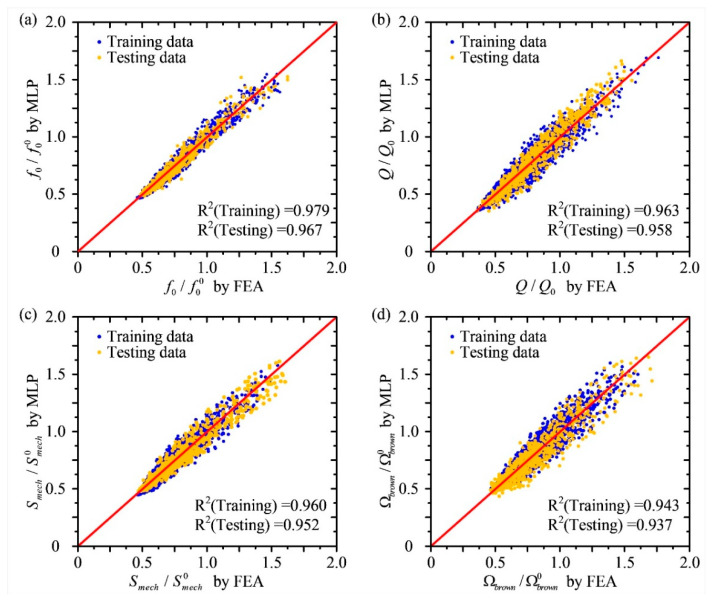
Predicted performances of automatically produced disk MEMS resonators given by the neural network analyzer based on the multilayer perceptron neural network compared with the traditional finite element analysis method. (**a**) The comparison with the fundamental frequency, where $f_{0}^{0}$ is normalized fundamental frequency reference; (**b**) the comparison with the quality factor, where *Q_0_* is normalized quality factor reference; (**c**) the comparison with the mechanical sensitivity, where $S_{mech}^{0}$ is normalized mechanical sensitivity reference; (**d**) the comparison with the mechanical thermal noise, where $\Omega_{brown}^{0}$ is normalized mechanical thermal noise reference.

In the process of evaluating the accuracy of the predictions produced by the multilayer perceptron neural network, the corresponding simulation results produced by the traditional finite element analysis method are chosen as the reference. The comparison between performance indicators calculated by the finite element analysis method and predicted by the multilayer perceptron neural network is depicted in Figure 8. In order to better characterize the cumulative error of the neural network, all the performance index parameters of the disk MEMS resonator are normalized. The accuracy of the multilayer perceptron neural network is defined by the mean squared error *E_k_*, as shown in Equation (17), which could be represented by the matching degree of the predictions from the neural network and the simulation results produced by the finite element analysis method. It is obvious that the training dataset and test dataset for different performance indicators both scatter around the straight line *y* = *x*. Moreover, the mean squared errors for different performance indicators of this neural network are all less than 0.06. It has been proved that the multilayer perceptron neural network has a high prediction accuracy and there is no overfitting problem.

Obviously, the neural network model after training has reached a very good accuracy and speed, which is of great significance for reducing repetitive work. Since the neural network is a learning model based on prior knowledge, it needs to provide certain data for its learning and training, so that the time cost of pre-training needs to be taken into consideration. Although it takes a certain amount of time for the neural network to grasp the internal characteristics of the resonator, this time is very short in a long time span. It can realize the fast simulation of the resonator’s performance under fixed constraints, greatly reducing the time-consuming and long repetitive work. In addition, the multilayer perceptron network also has great application potential in other fields. For the multilayer perceptron network, it does not care about the internal physical connection, but focuses on the connection between inputs and outputs. Therefore, for the problem of clarifying the input and output of the system, the multilayer perceptron neural network can obtain an ideal system architecture through learning and training with sufficient sample data [27,28,29,30,31,32]. It has important significance and value for the optimization and simulation of structural parameters in specific scenarios, and greatly improves design efficiency.

## 5. Conclusions

The complete procedures of the multilayer perceptron neural network have been discussed in detail in this paper. To provide enough samples, an automatic structural parameter generator is applied to produce different data within reasonable regions. The error back propagation algorithm is used to update the connection weights and thresholds in the neural network. The time consumption for optimizing the neural network structure is about 516 min after iterating 10^5^ dealing with 4000 samples. It is worth noting that the cumulative error of the multilayer perceptron neural network decreases to 0.0017 in the end, proving that the training performance of the neural network reaches a satisfying level.

This study has proven that when dealing with the same samples, the time consumption of the neural network is less than 1/15,000 of the time required for the finite element analysis, exhibiting excellent calculation characteristics. Moreover, the prediction accuracy of the neural network remains a remarkable level after an adequate training process. Compared with the traditional finite element analysis method, the mean squared errors for different performance indicators of this neural network are all less than 0.06. As a result, it can be concluded that the predication tool based on the multilayer perceptron neural network achieves a high speed and accuracy level.

The high-speed and high-accuracy mathematical modeling technology is a product of the artificial neural network in the traditional MEMS resonator design process, and it has great potential in enhancing the designing performance. It has been proved that it is a good method to accelerate the predication speed while considering the accuracy. Moreover, it is demonstrated that the artificial neural network model can provide a promising tool for the design and optimization of structures. This mathematical modeling method presented in this work is suitable for other MEMS devices, which can be applied in the optimization design after suitable training process.

## Figures and Tables

**Table 1:** The structural parameter range table of the disk MEMS resonator.

Structural Parameter	Symbol	Value Range	Unit
Height	*H*	100:100:500	μm
Anchor radius	*r*	1.5:0.5:5	mm
Ring number	*N*	5:1:20	1
Ring width	*Rw*	3:2:30	μm
Ring gap	*G*	100:20:600	μm
Spoke width	*Sw*	3:2:30	μm

**Table 2:** The core performance indicators table of the disk MEMS resonator.

Core Performance Indicators	Symbol	Unit
Fundamental frequency	*f_0_*	Hz
Quality factor	*Q*	1
Mechanical sensitivity	*S_mech_*	m/(°/s)
Mechanical thermal noise	Ω*_brown_*	°$/\sqrt{h}$

**Table 3:** The working flow chart of the error back propagation algorithm used in this paper.

**Input:**		$\mathrm{The}\;\mathrm{training}\;\mathrm{dataset}\;D={\left\{\left(x_{k},\;y_{k}\right)\right\}}_{k=1}^{m}\;\mathrm{and}\;\mathrm{the}\;\mathrm{learning}\;\mathrm{rate}\;\eta .$
**Process:**	1	Randomly initialize all connection weights and thresholds in the network within the range of (0, 1).
	2	**repeat**
	3	**for all ** $\left(x_{k},y_{k}\right)\in D$ ** do**
	4	$\mathrm{Calculate}\;\mathrm{the}\;\mathrm{output}\;\mathrm{value}\;{\hat{y}}_{k}$ of the current sample according to the current parameters and Equation (13).
	5	Calculate the gradient index *g_j_* of the neurons in the output layer according to Equation (19).
	6	Calculate the gradient index *e_h_* of the hidden layer neuron according to Equation (24).
	7	Update the connection weights *w_hj_*, *v_ih_* and thresholds *θ_j_*, *γ_h_* in the neural network according to Equations (20)–(23).
	8	**end for**
	9	**until** satisfies the stop condition (the cumulative error is less than 10^−3^ or the number of iterations exceeds 10^5^).
**Output:**		Multi-layer perceptron neural network after optimizing connection weights and thresholds.

## Data Availability

Data are contained within the article.
